# Pharmacovigilance of Androgen Receptor Pathway Inhibitors in Prostate Cancer: The Impact of Docetaxel Co-Reporting †

**DOI:** 10.3390/cancers18183049

**Published:** 2026-09-20

**Authors:** Onur Alkan, İsmail Nazlı, Ahmet Başgöze, Mahmut Gümüş

**Affiliations:** Department of Medical Oncology, Istanbul Medeniyet University, Göztepe Prof. Dr. Süleyman Yalçın City Hospital, Istanbul 34722, Türkiye; ismail.nazli@medeniyet.edu.tr (İ.N.); ahmet.basgoze@medeniyet.edu.tr (A.B.); mahmut.gumus@medeniyet.edu.tr (M.G.)

**Keywords:** androgen receptor pathway inhibitor, pharmacovigilance, prostate cancer, FAERS, docetaxel, confounding, adverse events

## Abstract

**Background/Objectives:** Androgen receptor pathway inhibitors (ARPIs) are widely used across prostate cancer disease states, but spontaneous-report safety signals may be distorted by co-administered therapies. We characterized ARPI-associated adverse event reporting in men aged 18–64 years with prostate cancer and tested whether robust signals persisted after accounting for documented docetaxel co-reporting. **Methods:** We analyzed deduplicated U.S. Food and Drug Administration Adverse Event Reporting System (FAERS) reports from 2014 Q1 through 2026 Q1 in which abiraterone, enzalutamide, apalutamide, or darolutamide was the primary suspect drug. Disproportionality was assessed using ROR, PRR, chi-square, and Bayesian information component metrics. Priority signals were tested across three reference backgrounds, followed by symmetric exclusion of docetaxel-co-reported cases from the darolutamide group and the comparator. **Results:** Among 19,814 age- and disease-restricted background reports, 4919 involved an ARPI as the primary suspect. Docetaxel was co-reported in 27.8% of darolutamide reports. Darolutamide-associated decreased neutrophil count remained positive across all three reference backgrounds and was IC025-supported, yet both decreased neutrophil count and myelosuppression fell to zero events after documented docetaxel exclusion. Peripheral neuropathy persisted with 20 events. Enzalutamide-associated fatigue and dizziness were more strongly reported in patients aged ≥ 65 years. **Conclusions:** ARPIs showed distinct adverse event reporting patterns in men aged 18–64 years with prostate cancer. Disappearance of darolutamide-associated hematologic signals after documented docetaxel exclusion highlights the value of regimen-level sensitivity analyses in pharmacovigilance of combination cancer therapies. Persistent peripheral neuropathy requires external evaluation because residual confounding from unreported or prior neurotoxic treatment cannot be excluded.

## Simple Summary

Androgen receptor pathway inhibitors (ARPIs), including abiraterone, enzalutamide, apalutamide, and darolutamide, are widely used across multiple stages of prostate cancer and have distinct safety profiles. Post-marketing pharmacovigilance is important for identifying adverse event reporting patterns that may not be fully characterized in clinical trials. However, safety signals in spontaneous-report databases can be difficult to interpret when anticancer drugs are routinely used in combination. We analyzed FDA Adverse Event Reporting System reports for these four ARPIs in men aged 18–64 years with prostate cancer. Darolutamide-associated decreased neutrophil count and myelosuppression remained statistically robust across multiple comparator groups but disappeared after reports containing documented docetaxel were excluded from both the darolutamide group and the comparator. These findings strongly support regimen-level confounding and highlight the importance of considering co-reported therapies when interpreting pharmacovigilance signals for combination cancer treatments.

## 1. Introduction

Prostate cancer is among the most commonly diagnosed malignancies in men worldwide, and the American Cancer Society projects approximately 333,830 new cases in the United States in 2026 [1,2]. Its development and progression are multifactorial and have been associated with a range of chemical, physical, and biological carcinogenic influences [3,4]. Although prostate cancer occurs predominantly in older men, an increasing proportion of diagnoses involve men younger than 65 years, for whom disease biology, treatment duration, and long-term functional outcomes may differ from those of older patients [5,6,7,8,9]. Androgen receptor pathway inhibitors (ARPIs)—abiraterone, enzalutamide, apalutamide, and darolutamide—have become standard components of treatment across multiple stages of prostate cancer [8,9,10,11,12,13,14]. Although they share a therapeutic target, their pharmacology and safety profiles differ. Abiraterone is associated with mineralocorticoid excess and hepatic toxicity; enzalutamide with central nervous system and functional adverse events; apalutamide with cutaneous toxicity; and darolutamide with comparatively low central nervous system penetration [15,16,17,18,19,20]. These differences may be particularly relevant to younger patients who may receive prolonged treatment and for whom cumulative functional toxicity can affect long-term quality of life.

FAERS provides a large post-marketing data source for detecting disproportionate reporting of adverse events. Previous pharmacovigilance analyses of ARPIs have described agent-specific cutaneous, cardiovascular, hepatobiliary, and neurological reporting patterns [21,22,23,24,25], while more recent work has incorporated geriatric or age-stratified comparisons [26,27,28]. Wu et al. identified multiple darolutamide-associated neurological and hematologic signals, including peripheral neuropathy, decreased neutrophil count, and myelosuppression, using several disproportionality algorithms [27]. Beypınar et al. likewise identified a prominent peripheral neuropathy signal with darolutamide and proposed concurrent docetaxel exposure as the most parsimonious explanation; however, their operational definition of ARPI monotherapy did not exclude concomitant non-ARPI therapies, and the contribution of docetaxel could not be directly resolved [28]. Neither analysis specifically tested whether the observed signals persisted after direct exclusion of documented docetaxel co-reporting. This illustrates a broader methodological problem in spontaneous-report pharmacovigilance: adverse events may be attributed to an index drug when they arise from a commonly co-administered component of the treatment regimen. Persistence of a signal across alternative comparator groups or supportive Bayesian shrinkage metrics establishes statistical robustness but does not necessarily establish drug specificity.

A preliminary analysis of age-restricted ARPI reporting using an earlier FAERS data cut and an OpenVigil-based extraction was presented at the 2026 ASCO Annual Meeting [29]. The present study substantially extends that work by processing raw quarterly FAERS files through 2026 Q1 with case-level deduplication, using an age-, sex-, and disease-restricted primary background, testing priority signals across alternative reference populations and Bayesian shrinkage metrics, and explicitly evaluating regimen-level confounding.

We therefore analyzed FAERS reports from 2014 Q1 through 2026 Q1 to characterize ARPI-associated adverse event reporting in men aged 18–64 years with prostate cancer and to determine whether apparently robust safety signals could be distinguished from regimen-level co-medication confounding. Because docetaxel was disproportionately co-reported with darolutamide, we performed a dedicated symmetric sensitivity analysis excluding docetaxel-co-reported cases from both the darolutamide group and its background comparator. An exploratory, false discovery rate-corrected comparison with reports from men aged ≥ 65 years was also performed to place the primary findings in an age-related context.

## 2. Materials and Methods

### 2.1. Study Design and Cohort Construction

We conducted a retrospective pharmacovigilance study using FAERS, a publicly available spontaneous reporting database containing adverse event reports submitted by healthcare professionals, consumers, and manufacturers [30,31,32]. Quarterly ASCII files from 2014 Q1 through 2026 Q1 were analyzed using the DEMO (demographic and administrative information), DRUG (reported drug exposure and drug role), REAC (adverse reactions coded as MedDRA preferred terms), INDI (reported treatment indications), and OUTC (reported clinical outcomes) tables. The study period began in 2014 Q1 to capture a contemporary ARPI pharmacovigilance era within a predefined common calendar window. Drugs introduced later contributed reports only after their respective market entry, resulting in different cumulative exposure periods across agents. Duplicate and follow-up submissions were resolved at the CASEID (unique case identifier) level by retaining the most recent case version, yielding 16,323,742 deduplicated FAERS cases in the full 2014 Q1–2026 Q1 source dataset before application of prostate cancer, sex, age, or ARPI-specific eligibility criteria.

Prostate cancer cases were identified using indication terms recorded in the INDI table containing “PROSTAT”, “CRPC”, “CASTRATION RESISTANT”, or “MALIGNANT NEOPLASM OF PROSTATE”. Analyses were restricted to reports coded as male to minimize potential sex-miscoding and improve cohort specificity. The primary cohort included reports in which abiraterone, enzalutamide, apalutamide, or darolutamide was coded as the primary suspect drug. The 18–64-year age range was selected a priori to focus on non-geriatric adults and to permit a separate exploratory comparison with patients aged ≥ 65 years. Patients were required to have a recorded age of ≥18 and <65 years. Reports with missing age or age ≥ 65 years were excluded from the primary analysis. Recorded age represents age at the time of reporting and not necessarily age at prostate cancer diagnosis.

### 2.2. Disproportionality Analysis

For each ARPI-adverse event pair, disproportionality was assessed within an age-, sex-, and disease-restricted FAERS background. The age-, sex-, and disease-restricted background was selected to improve clinical comparability between index-drug and comparator reports and to reduce heterogeneity related to demographic differences and confounding by indication that could arise when using the entire FAERS database as the primary reference population. Each index ARPI was compared with all other reports in the same background in which that drug was not coded as the primary suspect; therefore, the primary comparator was not restricted to the other three ARPIs. Standard 2 × 2 contingency tables were constructed for each drug–event pair.

Reporting odds ratios (RORs) with 95% confidence intervals (CIs), proportional reporting ratios (PRRs), and chi-square statistics were calculated. A pharmacovigilance signal was defined as at least three events in the index-drug group, PRR ≥ 2, chi-square ≥ 4, and a lower 95% confidence bound for the ROR > 1. An information component (IC) and its lower 95% credibility bound (IC025) were additionally calculated using a Bayesian shrinkage estimator with a continuity correction for sparse cells; IC025 > 0 was considered supportive evidence and did not replace the primary signal definition. No additional correction for multiple comparisons was applied to the primary disproportionality screens, consistent with their signal-detection purpose.

### 2.3. Prespecified and Exploratory Adverse Event Analyses

The primary analysis evaluated a prespecified set of clinically expected or biologically plausible MedDRA version 28.0 Preferred Terms (PTs) based on established ARPI safety profiles [33]. Related PTs were additionally grouped into clinically defined domains to assess whether individual drug-event associations were reflected in broader toxicity patterns.

An exploratory screen was subsequently performed across all reported PTs for each ARPI. Terms that did not represent interpretable adverse drug reactions—including product issues, dose omission, underdose, treatment discontinuation, lack of therapeutic effect, noncompliance, disease progression, hospitalization, and death—were removed using a prespecified noise dictionary. Findings based on small event counts were considered hypothesis-generating.

### 2.4. Comparator Robustness Analyses

To evaluate the influence of comparator selection, priority drug–event pairs were re-evaluated using three reference populations: (1) the primary age-, sex-, and prostate cancer-restricted background; (2) an internal ARPI-class background; and (3) the complete deduplicated FAERS database. Classical signal status and IC025 support were compared across these backgrounds. These analyses evaluated statistical stability across alternative comparator populations rather than causality or drug specificity.

An additional indication-free sensitivity cohort included all male patients aged 18–64 years in whom an ARPI was coded as the primary suspect drug, irrespective of the recorded indication. This analysis assessed whether key findings were dependent on restriction to reports explicitly coded for prostate cancer.

### 2.5. Docetaxel Co-Reporting and Regimen-Level Sensitivity Analysis

Docetaxel co-reporting was quantified in the primary background population and separately for each ARPI. Because docetaxel co-reporting was markedly concentrated among darolutamide reports and several observed darolutamide-associated signals were clinically compatible with taxane-related toxicity, a dedicated regimen-level sensitivity analysis was performed.

Reports containing documented docetaxel exposure were excluded symmetrically from both the darolutamide index group and its corresponding background comparator before reconstruction of the 2 × 2 contingency tables. The symmetric design avoided altering only the exposure group while leaving the reference population unchanged. This analysis was not applied uniformly to the other ARPIs because documented docetaxel co-reporting was uncommon in those groups. Darolutamide was additionally of particular interest because it has a regulatory-approved concurrent combination indication with docetaxel in metastatic hormone-sensitive prostate cancer [14]. Cabazitaxel and platinum-based chemotherapy were not selected for analogous exclusion because they are not routinely co-administered concurrently with ARPIs in standard practice.

The purpose of this analysis was to determine whether apparently robust darolutamide-associated reporting signals remained after accounting for documented co-reporting of a strongly correlated regimen component. Because spontaneous reports may incompletely capture concomitant or prior therapies, absence of documented docetaxel was not interpreted as proof of absence of taxane exposure. Detailed operational definitions for prostate cancer indication matching and ARPI/docetaxel identification are provided in Table S10.

### 2.6. Exploratory Age-Stratified Comparison

An exploratory comparison was performed between patients aged 18–64 years and those aged ≥ 65 years. Within each age stratum, each index ARPI was compared with non-index reports from the corresponding age- and disease-restricted background. Differences between log-transformed RORs were tested using z statistics derived from the corresponding 2 × 2 tables, and Benjamini–Hochberg false discovery rate correction was applied. Sparse-cell findings were interpreted cautiously regardless of nominal or FDR-adjusted statistical significance.

## 3. Results

### 3.1. Study Population

Among 16,323,742 deduplicated FAERS cases, 175,150 reports included a male prostate cancer indication and 19,814 involved patients aged 18–64 years. The primary cohort comprised 4919 reports in which an ARPI was the primary suspect: enzalutamide, 2688 (54.6%); abiraterone, 1547 (31.5%); apalutamide, 360 (7.3%); darolutamide, 324 (6.6%). The sequential construction of the primary study cohort is summarized in Table 1.

### 3.2. Prespecified and Exploratory Safety Signals

Eleven prespecified ARPI–event pairs met the combined signal criteria. Abiraterone was associated with hypokalemia (*n* = 23; ROR 6.11, 95% CI 3.69–10.13), drug eruption (*n* = 5; ROR 5.38, 95% CI 1.87–15.51), and increased aspartate aminotransferase (*n* = 11; ROR 2.21, 95% CI 1.16–4.22). For enzalutamide, seizure was the only prespecified signal (*n* = 24; ROR 2.23, 95% CI 1.40–3.55). Apalutamide signaled for rash (*n* = 34; ROR 6.48, 95% CI 4.47–9.39), hypertension (*n* = 15; ROR 3.01, 95% CI 1.77–5.12), increased liver function test (*n* = 4; ROR 7.53, 95% CI 2.63–21.52), and increased alanine aminotransferase (*n* = 7; ROR 3.96, 95% CI 1.82–8.58). Darolutamide signaled for rash (*n* = 14; ROR 2.64, 95% CI 1.53–4.56), asthenia (*n* = 26; ROR 2.11, 95% CI 1.40–3.17), and increased alanine aminotransferase (*n* = 6; ROR 3.73, 95% CI 1.63–8.58).

**Figure 1:**
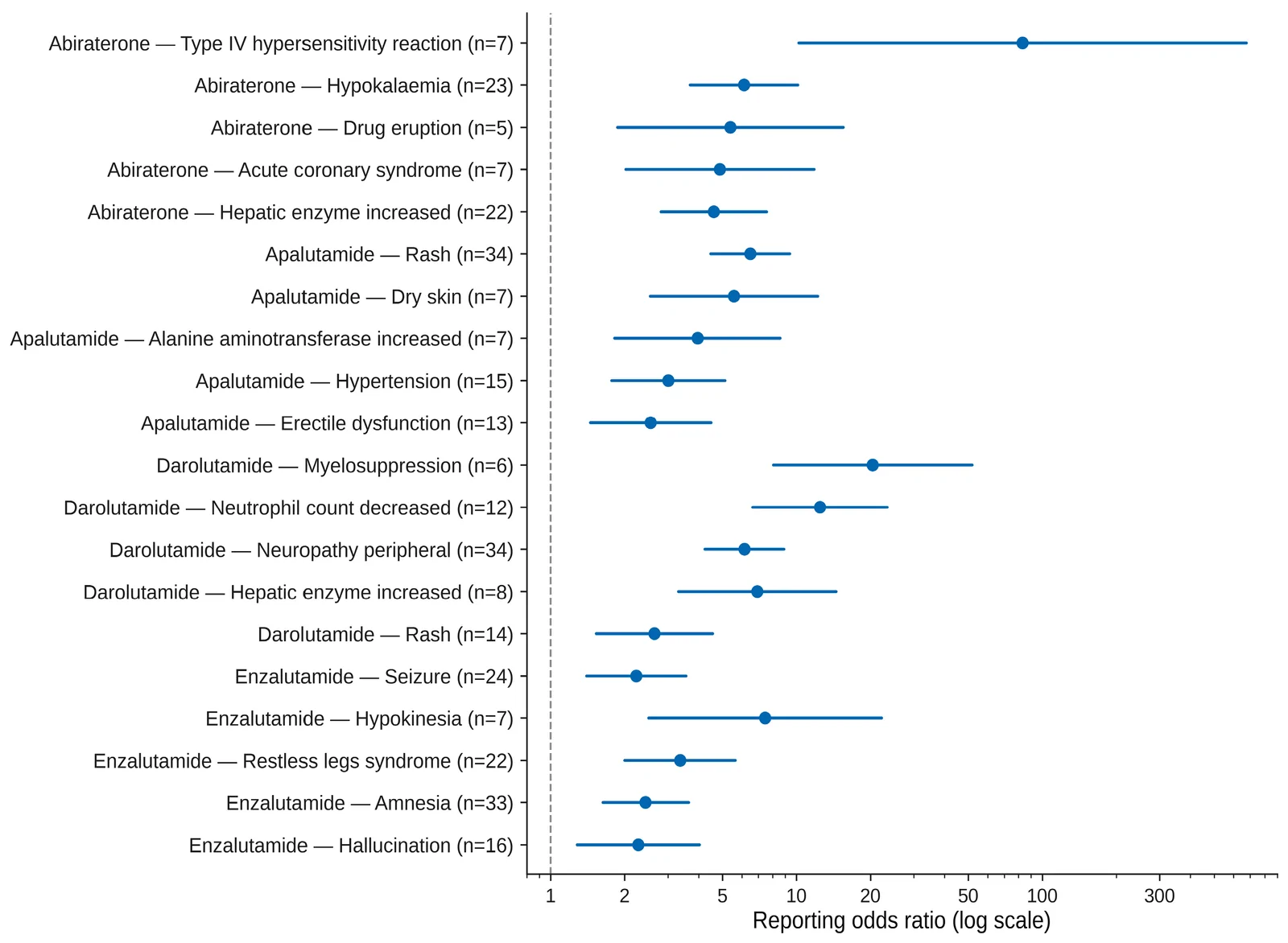
Selected exploratory all-Preferred-Term clinical signals for androgen receptor pathway inhibitors. Points represent reporting odds ratios, and horizontal bars indicate 95% confidence intervals.

**Figure 2:**
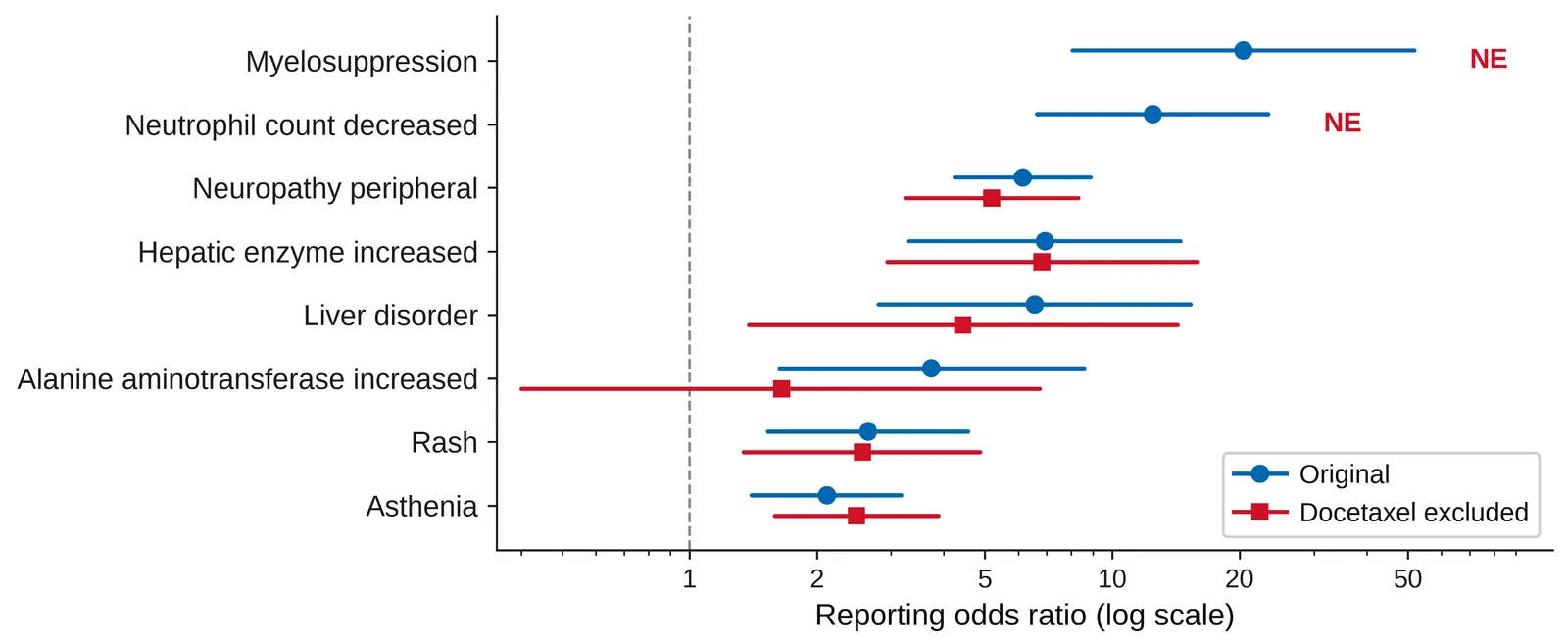
Darolutamide–docetaxel sensitivity analysis. Original and docetaxel-excluded reporting odds ratios are shown for selected darolutamide-associated adverse events. NE, not estimable because no events remained after exclusion of docetaxel-co-reported cases.

**Figure 3:**
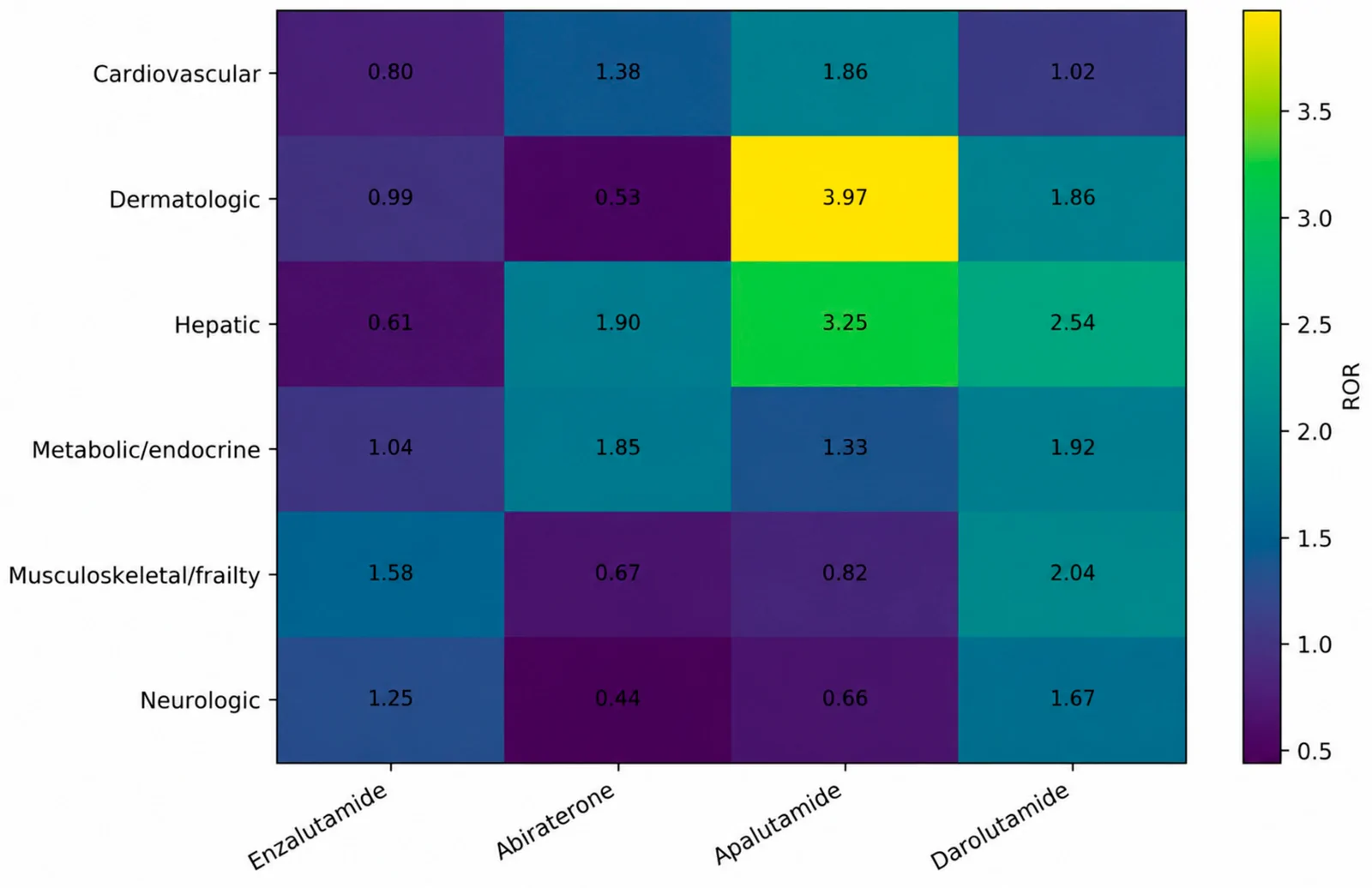
Overview of adverse event reporting patterns for ARPIs in the FAERS database.

At the domain level, apalutamide showed dermatological and hepatic signals, whereas darolutamide showed a hepatic signal. The filtered all-PT screen was concordant with the principal profiles: metabolic/hepatic events for abiraterone, neurological and functional events for enzalutamide, cutaneous events for apalutamide, and hepatic, cutaneous, hematologic, and neuropathic events for darolutamide. Selected exploratory all-PT findings are summarized in Figure 1, whereas the darolutamide–docetaxel sensitivity analysis is presented in Figure 2. An overview of domain-level reporting patterns is shown in Figure 3.

### 3.3. Darolutamide–Docetaxel Sensitivity Analysis

Docetaxel was co-reported in 1102/19,814 (5.56%) background reports and 195/4919 (3.96%) ARPI primary-suspect reports. Co-reporting was markedly concentrated in the darolutamide group: 90/324 (27.78%), compared with 40/1547 (2.59%) for abiraterone, 63/2688 (2.34%) for enzalutamide, and 2/360 (0.56%) for apalutamide.

After symmetric exclusion of docetaxel-co-reported cases, the background decreased from 19,814 to 18,712 reports and the darolutamide group from 324 to 234 reports. Myelosuppression and decreased neutrophil count both fell to zero events in the darolutamide arm and were no longer estimable. In contrast, peripheral neuropathy persisted with 20 events, and hepatic enzyme increased, liver disorder, rash, and asthenia remained signal-positive; increased alanine aminotransferase no longer met the signal criteria (Figure 2).

### 3.4. Robustness and Indication-Free Analyses

Abiraterone-associated hypokalemia and apalutamide-associated rash remained positive against the disease-restricted, ARPI class-internal, and all-FAERS backgrounds and were supported by IC025. Darolutamide-associated peripheral neuropathy and asthenia were also retained across backgrounds with IC025 support. Hepatic enzyme increases remained positive by classical criteria but lacked IC025 support. The robustness of priority signals across the three reference backgrounds, together with IC025 support, is summarized in Table 2.

Before docetaxel exclusion, decreased neutrophil count with darolutamide was positive across all three backgrounds and IC025-supported in each. Its complete disappearance after removal of docetaxel-co-reported cases strongly suggested that comparator variation and supportive IC metrics alone were insufficient to address regimen-level co-medication confounding. Enzalutamide-associated seizure remained positive in the disease-restricted and class-internal analyses but not against the all-FAERS or indication-free backgrounds and lacked IC025 support, indicating a label-consistent but comparator-sensitive finding. Sparse signals, including abiraterone drug eruption and selected apalutamide hepatic and severe cutaneous terms, were not consistently IC025-supported and were considered hypothesis-generating.

Removing the prostate cancer indication requirement increased the indication-free cohort from 4919 to 6861 reports. The central signals for abiraterone hypokalemia, apalutamide rash and hypertension, and darolutamide asthenia and peripheral neuropathy persisted. Enzalutamide fatigue, dizziness, and memory impairment, which did not meet criteria in the primary cohort, became positive and IC025-supported in this larger sensitivity cohort.

### 3.5. Age Availability and Exploratory Age Comparison

Of all ARPI primary-suspect reports, 4919 (8.49%) were aged 18–64 years, 33,233 (57.33%) were aged ≥ 65 years, 19,804 (34.16%) had missing age, and 13 (0.02%) were aged < 18 years. Reports with recorded adult age were more frequently coded as serious than those with missing age (72.92% vs. 57.75%) and more frequently included hospitalization (28.40% vs. 14.94%), suggesting non-neutral selection associated with age availability.

Most prespecified signals were shared across age strata or did not differ significantly after false discovery rate correction. Enzalutamide-associated dizziness (ROR ratio for 18–64 vs. ≥65 years, 0.65; 95% CI 0.53–0.80; q = 0.001) and fatigue (ROR ratio 0.71; 95% CI 0.63–0.81; q < 0.001) were more strongly reported in patients aged ≥ 65 years. An apparent age difference for apalutamide-associated increased alanine aminotransferase depended on only one event in the older group and was considered a sparse-cell artifact. Selected comparisons are shown in Table 3. Additional methodological definitions, complete prespecified and exploratory results, comparator analyses, docetaxel sensitivity analyses, missing-age characterization, and indication-free sensitivity results are provided in Tables S1–S10.

## 4. Discussion

### 4.1. Principal Findings and ARPI-Specific Reporting Patterns

Using an age-, sex-, and disease-restricted FAERS background, this study identified distinct and largely label-consistent reporting patterns for four major ARPIs in men aged 18–64 years with prostate cancer. The principal contribution, however, extends beyond description of drug-specific signals. Our findings demonstrate that a reporting association may remain statistically robust across multiple reference populations and receive supportive Bayesian shrinkage evidence while still potentially reflecting a co-administered component of the treatment regimen. Darolutamide-associated decreased neutrophil count was strongly disproportionate across the disease-restricted, ARPI class-internal, and all-FAERS backgrounds and was supported by IC025 in each analysis, yet disappeared completely after reports with documented docetaxel co-reporting were excluded. This distinction between statistical robustness and drug specificity is particularly relevant to pharmacovigilance of modern combination cancer therapies.

Abiraterone-associated hypokalemia was the clearest metabolic signal and remained robust across sensitivity analyses. This finding is consistent with CYP17A1 inhibition, adrenocorticotropic hormone-driven mineralocorticoid excess, and the increased rates of hypokalemia and hypertension reported in LATITUDE [8,15]. Severe manifestations, including hypokalemia-associated torsades de pointes, have been described [34]. Hepatic laboratory signals were also compatible with the established safety profile and prior pharmacovigilance studies [22,23]. Low-count findings such as drug eruption and selected QT-related terms should be interpreted cautiously because of limited event counts and inconsistent IC025 support.

Apalutamide demonstrated the strongest and most consistent dermatologic reporting pattern. Rash remained strongly disproportionate across alternative reference backgrounds and was supported by IC025, in agreement with SPARTAN and TITAN [11,12], reports of severe cutaneous reactions [18,19], and subsequent real-world pharmacovigilance analyses [21,24,25]. Hepatic and hypertension signals were also clinically plausible but were less stable when event counts were small.

For enzalutamide, seizure met signal criteria in the disease-restricted and ARPI class-internal analyses but attenuated when the comparator was expanded to the complete FAERS database or the indication-free population. This comparator dependence does not establish absence of risk; rather, it illustrates the sensitivity of disproportionality estimates to the background frequency of events in the selected reference population. The association remains biologically and clinically plausible because enzalutamide crosses the blood–brain barrier and can inhibit GABA-gated chloride channels [16]. Enzalutamide-associated fatigue and dizziness were more strongly reported among patients aged ≥ 65 years, consistent with recognized central nervous system and functional tolerability concerns [9,10,17,35]. These age comparisons reflect differential reporting rather than differences in absolute clinical incidence or comparative drug risk.

### 4.2. Darolutamide–Docetaxel Co-Reporting and Regimen-Level Confounding

The most informative findings arose from the darolutamide-docetaxel analysis. Docetaxel was co-reported in 27.78% of darolutamide reports, compared with 5.56% of the disease-restricted background. After symmetric exclusion of docetaxel-co-reported cases from both the darolutamide and reference populations, decreased neutrophil count and myelosuppression fell to zero events in the darolutamide group. Decreased neutrophil count had previously met conventional signal criteria across all three reference backgrounds and was IC025-supported in each. Thus, neither comparator variation nor Bayesian shrinkage identified the regimen-level origin of this apparently robust reporting association.

These findings are clinically coherent with ARASENS, in which grade 3–4 neutropenia was similar in the darolutamide and placebo groups receiving docetaxel and most common adverse events occurred during the overlapping chemotherapy period [14]. Taken together, these observations strongly support regimen-level confounding and argue against interpreting the hematologic signals as direct darolutamide toxicity; however, FAERS cannot establish which component of the regimen caused these events.

A recent darolutamide-focused FAERS analysis by Wu et al. independently identified peripheral neuropathy (ROR 10.56), decreased neutrophil count (ROR 7.17), and myelosuppression (ROR 6.91), with support from multiple disproportionality algorithms, including Bayesian methods [27]. These findings provide external concordance with the unadjusted darolutamide signals observed in our study. However, regimen-level docetaxel co-reporting was not specifically evaluated in that analysis. In the present study, the two hematologic signals disappeared completely after symmetric exclusion of documented docetaxel-associated reports, despite their initial robustness across multiple reference backgrounds and Bayesian support. Taken together, these findings illustrate that reproducibility across pharmacovigilance analyses and statistical algorithms does not by itself establish drug specificity when a strongly correlated co-medication is present.

Beypınar et al. recently identified a strong peripheral neuropathy signal with darolutamide and proposed concomitant docetaxel exposure in ARASENS-type treatment as the most parsimonious explanation [28]. Their operational definition of ARPI monotherapy, however, reflected the presence of a single suspect ARPI and did not exclude concomitant non-ARPI therapy; the potential contribution of docetaxel therefore could not be directly resolved. The present analysis extends this observation by explicitly evaluating documented docetaxel co-reporting at the case level.

Peripheral neuropathy behaved differently from the hematologic signals. Although the number of darolutamide-associated peripheral neuropathy reports decreased after removal of documented docetaxel co-reporting, 20 events remained and the signal persisted. This demonstrates that documented concurrent docetaxel exposure did not fully account for the observed reporting pattern, but it does not establish a causal relationship between darolutamide and peripheral neuropathy.

### 4.3. Persistent Peripheral Neuropathy and Residual Confounding

Residual confounding remains an important alternative explanation. Concomitant medications are incompletely captured in spontaneous reports, and cases classified as docetaxel-free may have involved undocumented concurrent docetaxel use. FAERS also cannot reliably reconstruct previous taxane exposure, cumulative treatment history, or exposure to other potentially neurotoxic agents. Some peripheral neuropathy reports may therefore reflect prior or unrecorded therapy rather than darolutamide itself, and non-treatment-related causes cannot be adequately assessed. The persistent signal should consequently be considered hypothesis-generating and warrants evaluation in datasets containing longitudinal treatment histories and more complete medication information.

The contrasting behavior of hematologic and neuropathic signals provides a broader methodological lesson. Alternative comparator populations address one source of instability in disproportionality analysis, and Bayesian shrinkage can reduce the influence of sparse observations, but neither approach inherently separates the effect of an index drug from that of a strongly correlated co-medication. In oncology, where doublet and triplet regimens are increasingly common, a statistically stable drug-event association may therefore represent a regimen-level signal rather than an index-drug effect. Explicit assessment of highly correlated co-medications should be considered when the clinical context provides a plausible alternative source for an observed safety signal.

### 4.4. Exploratory Age-Stratified Findings

The exploratory comparison between patients aged 18–64 years and those aged ≥ 65 years provided additional context but was not the primary basis for the study’s conclusions. Most prespecified signals were either shared between age strata or did not differ significantly after false discovery rate correction. Enzalutamide-associated dizziness and fatigue were more strongly reported in the older group, whereas an apparent age difference for apalutamide-associated alanine aminotransferase elevation was based on only one event in the older stratum and was therefore interpreted as a sparse-cell artifact.

### 4.5. Strengths, Limitations, and Future Directions

The study has several strengths. Reports were deduplicated at the CASEID level using complete quarterly FAERS files rather than relying on aggregate dashboard outputs. The primary comparator was restricted by age, sex, and disease; priority signals were subsequently examined across alternative reference populations and with Bayesian shrinkage metrics. Prespecified and exploratory PT analyses, an indication-free sensitivity cohort, an FDR-corrected age comparison, and a symmetric docetaxel exclusion analysis were used to examine different sources of signal instability. The analysis of missing age also demonstrated that reports with recorded age were enriched for serious outcomes, making an important source of selection bias explicit.

Several limitations should be considered. FAERS lacks exposure denominators and therefore cannot provide incidence rates, absolute risks, or comparative safety estimates. Spontaneous reports are subject to underreporting, stimulated reporting, notoriety bias, incomplete clinical information, and residual duplicate or follow-up reporting despite case-level deduplication [30,31,32,36,37,38]. Confounding by indication and co-medication cannot be fully controlled because disease stage, treatment line, performance status, cumulative exposure, prior therapy, and many comorbidities are incompletely or inconsistently recorded. Missing age excluded a substantial proportion of ARPI reports and was associated with differences in seriousness and hospitalization coding, indicating that the age-restricted cohort may not be representative of all exposed patients. Approval dates and cumulative market exposure differed across agents and may also have influenced report volumes. Low-count signals and the exploratory all-PT screen remain vulnerable to false-positive findings, and IC025 was used as supportive rather than confirmatory evidence. Most importantly, the docetaxel sensitivity analysis accounted only for documented co-reporting. Failure to record concomitant or previous docetaxel exposure may result in residual misclassification, particularly for peripheral neuropathy. Consequently, persistence of a signal after exclusion of documented docetaxel should not be interpreted as evidence that the event is directly attributable to darolutamide.

Future studies should validate the priority signals identified here using longitudinal data sources, including electronic health records, claims databases, and clinical registries that provide treatment timing, duration, cumulative exposure, prior taxane therapy, disease stage, and relevant comorbidities. Such datasets would be particularly valuable for evaluating the persistent peripheral neuropathy signal and for distinguishing effects attributable to the index ARPI from those related to concurrent or previous therapies.

## 5. Conclusions

ARPIs demonstrated distinct and largely label-consistent adverse event reporting patterns in men aged 18–64 years with prostate cancer. Darolutamide-associated decreased neutrophil count and myelosuppression were initially robust across multiple reference backgrounds but disappeared after exclusion of documented docetaxel co-reporting, strongly supporting regimen-level confounding and arguing against interpretation as direct darolutamide toxicity; however, FAERS cannot establish which component of the regimen caused these events. In contrast, peripheral neuropathy remained signal-positive after docetaxel exclusion, although residual confounding from unreported or prior neurotoxic therapy cannot be excluded. These findings highlight the importance of evaluating co-reported therapies when interpreting pharmacovigilance signals for anticancer agents commonly used in combination and support external validation of persistent signals in longitudinal datasets with detailed treatment histories. Because age was missing in 34.2% of ARPI primary-suspect reports, the 18–64-year cohort represents a selected subset of FAERS reports; these findings should not be interpreted as age-specific incidence or comparative safety estimates.

## Figures and Tables

**Table 1:** Construction of the primary study cohort.

Cohort Selection Step	*n*
Deduplicated FAERS cases, 2014 Q1–2026 Q1	16,323,742
Male prostate cancer indication reports	175,150
Male prostate cancer reports aged 18–64 years	19,814
ARPI primary-suspect reports	4919
Enzalutamide	2688
Abiraterone	1547
Apalutamide	360
Darolutamide	324

**Table 2:** Robustness of priority signals across three reference backgrounds, with IC025 support.

ARPI—Preferred Term	*n*	Disease-Restricted ROR (95% CI)	Class-Internal ROR (95% CI)	All-FAERS ROR (95% CI)	IC025 (DR/CI/AF)
Abiraterone—Hypokalemia	23	6.11 (3.69–10.13)	12.71 (4.39–36.81)	7.43 (4.92–11.21)	0.71/0.28/1.10
Abiraterone—Hepatic enzyme increased	22	4.61 (2.81–7.56)	4.85 (2.29–10.27)	4.55 (2.99–6.94)	0.51/0.04/0.71
Apalutamide—Rash	34	6.48 (4.47–9.39)	6.89 (4.50–10.55)	4.91 (3.45–6.99)	1.17/1.00/1.00
Darolutamide—Neuropathy peripheral	34	6.14 (4.24–8.89)	8.30 (5.39–12.79)	24.66 (17.28–35.18)	1.13/1.14/2.04
Darolutamide—Asthenia	26	2.11 (1.40–3.17)	2.23 (1.45–3.42)	5.02 (3.36–7.50)	0.02/0.02/0.86
Darolutamide—Neutrophil count decreased	12	12.46 (6.63–23.38)	22.05 (8.95–54.35)	19.80 (11.12–35.24)	0.57/0.54/0.69
Darolutamide—Myelosuppression	6	20.41 (8.05–51.76)	17.32 (5.26–57.06)	9.83 (4.38–22.04)	≤0/≤0/≤0
Apalutamide—Hypertension	15	3.01 (1.77–5.12)	2.83 (1.60–5.00)	4.53 (2.70–7.59)	0.04/≤0/0.38
Darolutamide—Hepatic enzyme increased	8	6.92 (3.31–14.50)	4.82 (2.15–10.82)	7.99 (3.96–16.11)	≤0/≤0/≤0
Darolutamide—Rash	14	2.64 (1.53–4.56)	2.31 (1.30–4.11)	2.13 (1.25–3.63)	≤0/≤0/≤0
Enzalutamide—Amnesia	33	2.43 (1.63–3.64)	5.53 (2.16–14.20)	4.68 (3.32–6.60)	0.15/≤0/1.00
Enzalutamide—Seizure	24	2.23 (1.40–3.55)	10.04 (2.37–42.53)	1.32 (0.88–1.98) NS	≤0/≤0/≤0
Abiraterone—Drug eruption	5	5.38 (1.87–15.51)	2.18 (0.63–7.55) NS	4.22 (1.75–10.15)	≤0/≤0/≤0
Apalutamide—Stevens–Johnson syndrome	4	11.49 (3.89–33.96)	17.06 (3.80–76.54)	15.04 (5.61–40.30)	≤0/≤0/≤0
Apalutamide—Liver function test increased	4	7.53 (2.63–21.52)	6.39 (1.92–21.33)	8.92 (3.33–23.91)	≤0/≤0/≤0

DR, disease-restricted 18–64-year male prostate cancer background; CI, ARPI class-internal background; AF, all-FAERS background; NS, not significant. Darolutamide neutrophil count decreased and myelosuppression met robustness criteria yet fell to zero events after symmetric exclusion of docetaxel-co-reported cases, strongly supporting regimen-level confounding and arguing against interpretation as direct darolutamide toxicity.

**Table 3:** Selected age-stratified prespecified comparisons: event counts, RORs, and interpretation.

ARPI	Preferred Term	18–64 *n*; ROR (95% CI)	≥65 *n*; ROR (95% CI)	ROR Ratio 18–64/≥65	FDR q	Interpretation
Abiraterone	Hypokalemia	*n* = 23; 6.11 (3.69–10.13)	*n* = 274; 7.19 (6.14–8.42)	0.85 (0.50–1.44)	0.816	Consistent in both ages; no age difference
Abiraterone	Drug eruption	*n* = 5; 5.38 (1.87–15.51)	*n* = 17; 1.35 (0.81–2.24)	4.00 (1.23–12.93)	0.134	Low N; no age difference after FDR
Apalutamide	ALT increased	*n* = 7; 3.96 (1.82–8.58)	*n* = 1; 0.12 (0.02–0.84)	33.71 (4.08–278.33)	0.023	Sparse-cell artifact; not interpreted as a robust age effect
Apalutamide	Rash	*n* = 34; 6.48 (4.47–9.39)	*n* = 354; 10.85 (9.58–12.30)	0.60 (0.40–0.88)	0.131	Strong in both ages; no age difference after FDR
Darolutamide	Asthenia	*n* = 26; 2.11 (1.40–3.17)	*n* = 76; 1.17 (0.92–1.47)	1.81 (1.13–2.89)	0.131	Signal at 18–64; no age difference after FDR
Enzalutamide	Seizure	*n* = 24; 2.23 (1.40–3.55)	*n* = 229; 3.36 (2.82–4.00)	0.66 (0.40–1.09)	0.332	Consistent in both ages; no age difference
Enzalutamide	Dizziness	*n* = 129; 1.34 (1.11–1.63)	*n* = 1234; 2.07 (1.93–2.22)	0.65 (0.53–0.80)	0.001	Stronger at ≥65; reliable given large N
Enzalutamide	Fatigue	*n* = 462; 2.02 (1.81–2.26)	*n* = 3400; 2.83 (2.71–2.97)	0.71 (0.63–0.81)	<0.001	Stronger at ≥65; reliable given large N
Enzalutamide	Memory impairment	*n* = 42; 1.90 (1.34–2.69)	*n* = 319; 3.10 (2.68–3.58)	0.61 (0.42–0.89)	0.131	Trend toward ≥65; no age difference after FDR

An ROR ratio < 1 indicates relatively stronger reporting in the ≥65-year group; >1 indicates stronger reporting in the 18–64-year group. Although the apalutamide ALT increased row was FDR-significant, it was based on only one event in the ≥65-year group and was interpreted as a sparse-cell artifact.

## Data Availability

The source data are publicly available from the U.S. Food and Drug Administration. The derived analytic datasets used in this study are available from the corresponding author on reasonable request.

## Supplementary Materials

The following supporting information can be downloaded at: https://www.mdpi.com/article/10.3390/cancers18183049/s1, Table S1: Exact/narrow exclusion dictionary used for the exploratory all-Preferred-Term screen; Table S2: Complete prespecified adverse event Preferred Term list and rationale; Table S3: Primary reference population and comparator definition; Table S4: Filtered clinical all-Preferred-Term signals; Table S5: Docetaxel co-reporting and darolutamide–docetaxel sensitivity analysis; Table S6: All prespecified Preferred Term-level results; Table S7: Priority signals across alternative reference backgrounds with IC025 support; Table S8: Missing-age bias characterization among ARPI primary-suspect reports; Table S9: Indication-free sensitivity analysis; Table S10: Operational definitions for prostate cancer indication and ARPI/docetaxel identification.
